# Rapid Onset of Type 1 Diabetes Mellitus and Destructive Thyroiditis Following Immunotherapy for Metastatic Melanoma

**DOI:** 10.1016/j.aed.2025.06.004

**Published:** 2025-06-25

**Authors:** Fatima Hallak, Gael Charbonne, Carrie Worley, Stephen McDonald

**Affiliations:** 1 Kettering Health Main Campus, Department of Internal Medicine, Kettering, Ohio; 2 Kettering Health Miamisburg, Kettering Health Medical Group Internal Medicine Clinic, Miamisburg, Ohio

**Keywords:** autoimmune endocrinopathies, destructive thyroiditis, immune checkpoint inhibitors, cancer immunotherapy, immune-related adverse events, type 1 diabetes mellitus

## Abstract

**Background:**

Immune checkpoint inhibitors, also called immunotherapy, enhance antitumor immunity but may also trigger autoimmune complications due to their mechanism of action. This is a case of rapid-onset multiple endocrinopathies, including type 1 diabetes mellitus and thyroiditis following immunotherapy.

**Case Report:**

A 57-year-old male with metastatic melanoma began immunotherapy with nivolumab and ipilimumab. Five weeks later, he was hospitalized with fatigue and abdominal pain. Blood glucose was 607 mg/dL, bicarbonate was 10 mmol/L (reference: 24-30 mmol/L), and arterial pH was 7.21 (reference: 7.35-7.45). Anion gap was 20 mmol/L (reference: 7-16 mmol/L), and beta-hydroxybutyrate was 6.48 mmol/L (reference: 0.02-0.27 mmol/L). His glutamic acid decarboxylase 65 antibody and insulin levels were elevated. His thyroid-stimulating hormone was <0.01 mIU/L (reference: 0.45-4.5 mIU/L), and free thyroxine was 3.1 ng/dL (reference: 0.88-1.77 ng/dL). He was treated for diabetic ketoacidosis and started on subcutaneous insulin. Immunotherapy was resumed at discharge. He later developed severe hypothyroidism requiring supplementation.

**Discussion:**

Immune checkpoint inhibitor-induced type 1 diabetes mellitus requires early recognition and management. Thyroid dysfunction is more common in these patients but can become severe and irreversible in the case of inflammatory thyroiditis. Genetic factors may predispose individuals to immune-related adverse events.

**Conclusion:**

Close vigilance is essential when using immunotherapy, as endocrinopathies can rapidly onset. In the future, genetic testing might help tailor immunotherapy and monitoring for such adverse events.

**Highlights**

• Immune-related endocrinopathies can emerge rapidly or after therapy cessation, with an average onset of 4 months
• They can be classified by severity using the Common Terminology Criteria for Adverse Events classification (stage 1-5), with most cases being stage 1 and 2
• The thyroid is the most commonly affected organ, ranging from subclinical to overt hypo- and hyperthyroidism and inflammatory thyroiditis
• If the endocrinopathy is manageable, checkpoint inhibitor therapy can be safely resumed in most cases
• Genetic profiles, including human leukocyte antigen genotyping, may predict the occurrence and type of immune-related adverse events

**Clinical Relevance**

This case emphasizes the need for vigilant monitoring of immune-related adverse events in patients receiving combination immune checkpoint inhibitors. Early recognition and appropriate management of endocrinopathies, including type 1 diabetes and thyroiditis, are crucial to ensuring continued immunotherapy efficacy while minimizing long-term complications.

## Introduction

Nivolumab and ipilimumab, immune checkpoint inhibitors (ICIs) targeting the programmed cell death protein 1 (PD-1) and cytotoxic T-lymphocyte antigen 4 (CTLA-4) pathways, respectively, play pivotal roles in modulating the immune system. This dual blockade enhances T-cell function and proliferation, amplifying the immune system's ability to target cancer cells. However, this heightened immunity can sometimes result in a variety of immune-related adverse events (irAEs), including endocrinopathies.^1^ These irAEs can occur due to lymphocytic infiltration, leading to tissue inflammation and damage, or due to the formation of autoantibodies. Dual immunotherapy is associated with a higher incidence of irAEs, which can affect various glands and manifest more rapidly and severely. While autoimmune thyroiditis, hepatitis, colitis, and pneumonitis are commonly observed, the occurrence of type 1 diabetes mellitus (T1DM) is less common.

## Case Report

A 57-year-old male presented with worsening fatigue and diffuse abdominal pain over the past week. He was afebrile and hemodynamically stable. Body mass index was 27.7 kg/m^2^. Thyroid examination revealed no enlargement, nodules, induration, or tenderness. Ophthalmologic examination was unremarkable.

He had a history of primary hypertension and no personal or family history of diabetes mellitus or autoimmune diseases. He had a history of melanoma that was diagnosed 5 years previously and had been surgically removed. Unfortunately, 5 weeks ago, multiple liver lesions indicative of metastatic melanoma were discovered. He began nivolumab and ipilimumab every 3 weeks, completing his second cycle 2 weeks before presentation.

On presentation, his blood glucose was 607 mg/dL, bicarbonate was 10 mmol/L (reference: 24-30 mmol/L), pH on arterial blood gas was 7.21 (reference: 7.35-7.45), anion gap was 20 mmol/L (reference 7-16 mmol/L) and beta-hydroxybutyrate was 6.48 mmol/L (reference: 0.02-0.27 mmol/L). His hemoglobin A1c was 6.3% (reference: 4%-5.6%), thyroid-stimulating hormone (TSH) <0.01 mIU/L (reference: 0.45-4.5 mIU/L), free thyroxine (T4) 3.1 ng/dL (reference: 0.88-1.77 ng/dL), glutamic acid decarboxylase 65 antibody (anti-GAD65) 122 U/mL (reference: <5 U/mL), insulin antibodies 12 uU/mL (reference: <5 uU/mL), negative anti-zinc transporter 8 antibodies, and C-peptide <0.1 ng/mL (reference: 1.1-4.4 ng/mL). Before immunotherapy, his A1c was 4.8%, hemoglobin was 15.9 g/dL, TSH was 2.1 mIU/L, and free T4 was 1.1 ng/dL, and before his second cycle, his TSH was 2.7 mIU/L and free T4 was 1.8 ng/dL. Computarized tomography abdomen showed no pancreatic abnormalities (Fig.). Neck ultrasound revealed a multinodular thyroid gland, which was new compared to previous surveillance computarized tomography imaging. He was managed with insulin drip and fluid resuscitation for diabetic ketoacidosis, and discharged on day 4 with insulin glargine 35 units nightly, 10 units of insulin lispro before meals, and a correction scale. Given that his endocrinopathies were deemed manageable, the oncology team resumed his dual immunotherapy regimen upon discharge.

**Fig:**
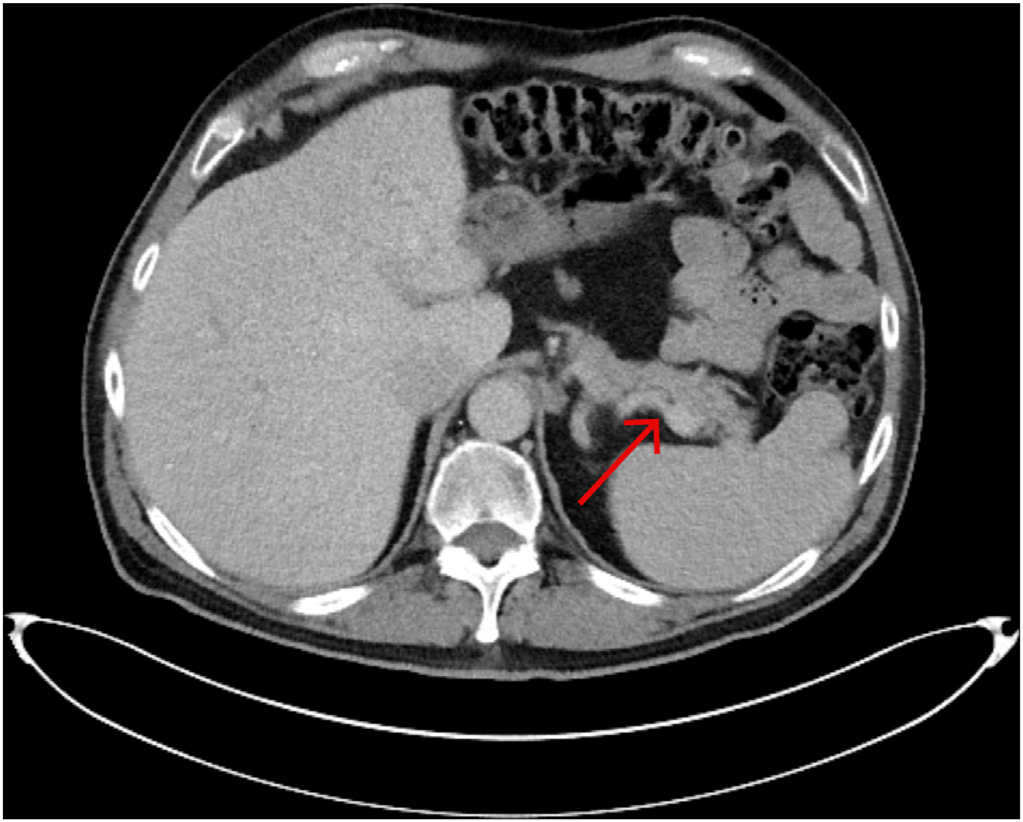
CT abdomen with intravenous contrast, transverse plane, the pancreas (red arrow) appears structurally normal without signs of inflammation or masses.

Early outpatient follow-up revealed persistent hyperglycemia requiring an increase in insulin regimen to glargine 46 units nightly, 12 units lispro premeals, and a moderate correction scale. At 2 months follow-up, his TSH was 18 mIU/L with a free T4 of 0.42 ng/dL. Morning serum cortisol was 12.3 μg/dL (reference: 6.7-22.6 μg/dL). Levothyroxine was initiated based on ideal body weight at 100 mcg daily. Further testing showed thyroid peroxidase (TPO) antibody of 11 (reference: 0-34 IU/mL), thyroid-stimulating hormone receptor antibody <1.10 IU/L (reference: <1.75 IU/L), and thyroid stimulating immunoglobulin <0.10 IU/L (reference: <0.10 IU/L). His TSH continued to rise, peaking at 50.3 mIU/L, for which levothyroxine was gradually increased to 175 mcg daily, leading to normalization of his TSH levels.

## Discussion

The patient was diagnosed with T1DM of autoimmune origin, supported by elevated anti-GAD65 antibodies. Additionally, he developed destructive thyroiditis, initially presenting with hyperthyroidism, which then progressed to hypothyroidism. Given the temporal association between the initiation of dual ICI and the onset of these endocrinopathies, we attributed the autoimmune manifestations to the recent immunotherapy.

This case supports growing evidence that while ICIs have revolutionized cancer treatment, they can also increase the risk of autoimmune reactions by enhancing the immune response. Endocrine irAEs are more common in women than in men, occurring about 4.1 ± 2.8 months after starting treatment, but they can also arise after therapy cessation.^2^^,^^3^ Combination therapy leads to earlier and higher incidence of endocrinopathies compared to single-agent regimens.

The severity of endocrinopathies is typically classified using the Common Terminology Criteria for Adverse Events (CTCAE), which ranges from grade 1 (asymptomatic or mild symptoms) to grade 5 (fatal).^4^ ICI-related endocrinopathies are predominantly grade 1 or 2, with the incidence of grade 3 and 4 cases being 0.68%.^5^

Current recommendations for monitoring include baseline testing of TSH, free thyroxine, morning cortisol, adrenocorticotropic hormone, and blood glucose before therapy initiation. Thyroid function should be reassessed every 6 to 8 weeks for the first 12 weeks. Adrenal function should be evaluated every 6 to 8 weeks if symptoms of adrenal insufficiency arise, and blood glucose should be monitored every 3 months in patients with diabetes risk factors.^6^

The thyroid gland is the most frequently affected organ, occurring in 8.5% to 11% of patients treated with monotherapy, and up to 16% with dual therapy, with subclinical thyrotoxicosis being the most prevalent presentation.^7^ In cases of hypothyroidism, it is essential to distinguish whether it is due to thyroiditis or hypopituitarism, as hypophysitis is also a common irAE.^7^ Hypophysitis is more common with CTLA-4 blockade, with an incidence of 0.4% to 17%, compared to <1% with PD-1 inhibitors. Adrenalitis is much less frequent (0.5%-2%) and typically presents later compared to other irAEs. T1DM is observed in 0.2% to 1.9% of patients on monotherapy and in up to 3.5% with combination therapy.^8^

A case series of ICI-induced T1DM highlighted both humoral and cellular mechanisms of β-cell destruction. Some patients had T1DM-associated autoantibodies, while others had diabetes-specific cluster of differentiation 8+ T cells, resembling findings in childhood-onset T1DM.^9^ About 50% of ICI-induced T1DM are associated with one or more islet autoantibodies, most commonly anti-GAD65, with anti-islet cell antibody, and anti-insulin autoantibody also frequently reported.^10^ Some cases documented the seroconversion of autoantibodies by comparing testing before and shortly after initiation of ICI.^11^ Some studies suggest that certain patients who develop ICI-induced T1DM may have been in an early, subclinical stage of latent autoimmune diabetes, with ICI therapy accelerating its onset. In one case, a polyclonal GAD65 antibody response, predominantly of the immunoglubulin G1 subclass, was detected just 5 days after initiating PD-1 inhibitor therapy. Given that IgG antibodies are typically involved in memory immune responses, the rapid onset of T1DM in this case implies that these antibodies were likely present before treatment and were merely amplified by the immunotherapy.^12^

One study found associations between specific HLA-DR alleles and various irAEs; 70% of patients who developed T1DM possessed the HLA-DR4 allele, 50% of those with hypothyroidism had HLA-DR8, and 63% of patients with hypophysitis carried HLA-DR15. These findings suggest that certain genetic profiles may influence the occurrence and type of irAEs, highlighting the potential of HLA genotyping before ICI therapy to identify patients at risk for specific adverse events and tailor their monitoring.^13^

In a 2019 meta-analysis, the median time to onset of T1DM after ICI therapy was 49 days, with diabetic ketoacidosis occurring in 76% of cases. The mean glycated hemoglobin A1c at presentation was 7.84 ± 1%. Otherwise, patients presented with hyperglycemia on routine testing, polyuria, fatigue, and excessive thirst. Notably, all the patients in that study necessitated lifelong insulin therapy.^14^

Most endocrine irAEs are irreversible regardless of discontinuation of the ICI and require long-term management. The risk of persistent dysfunction is significantly increased in the presence of organ-specific autoantibodies and when dual immunotherapy is used. While hyperthyroidism was transient in most cases, thyroid-stimulating hormone receptor antibody positivity was associated with persistent thyrotoxicosis. Patients with positive TPO antibodies had 11.6-fold higher odds of persistent hypothyroidism compared to TPO-negative individuals. All patients who developed thyroid irAEs on dual immunotherapy experienced persistent dysfunction. Pituitary irAEs were also predominantly irreversible, and their onset occurred relatively early during treatment. For T1DM, all cases were permanent.^8^

Unlike other irAEs, autoimmune endocrinopathies are often manageable with appropriate intervention. For patients with CTCAE grade 1 endocrinopathies, immunotherapy can be continued. However, immunotherapy should be paused for CTCAE grade 2 to 4 endocrinopathies until the condition is properly managed.^6^ The risk of developing new irAEs does not increase when PD-1 inhibitors are resumed in patients who have recovered from grade 1 to 3 irAEs.^15^ The safety of restarting immunotherapy after grade 4 irAEs remains uncertain, and further research is needed.

## Conclusion

Regular endocrine monitoring is crucial for patients undergoing ICI therapy to enable early detection of complications. Effective management of irAEs depends on close collaboration between endocrinologists and oncologists to maintain the therapeutic benefits of ICIs when feasible. Looking ahead, integrating genetic testing may support personalized ICI selection, tailored monitoring, and decreased complication rates, ensuring safer and more individualized immunotherapy.

## Statement of patient consent

Written informed consent was obtained from the patient for the publication of this case report.

## Disclosure

The authors have no conflicts of interest to disclose.
